# Calcium hexa­kis(dihydrogen­phosphito)­stannate(IV), Ca[Sn(H_2_PO_2_)_6_], with some remarks on the so-called Ge_2_(H_2_PO_2_)_6_ structure type

**DOI:** 10.1107/S1600536813018205

**Published:** 2013-07-06

**Authors:** Tobias Gieschen, Hans Reuter

**Affiliations:** a Institut für Chemie neuer Materialien, Strukturchemie, Universität Osnabrück, Barbarastr. 7, D-49069 Osnabrück, Germany

## Abstract

The title compound, Ca[Sn(H_2_PO_2_)_6_], was formed after a few days when tin(II) fluoride was allowed to react with phosphinic acid at ambient conditions. The structure consists of chains of Ca^2+^ and Sn^4+^ cations in octa­hedral sites with -3 symmetry bridged by bidentate hypophosphite anions. The chains are hexa­gonally close packed along [001]. The discovery of the compound and the successful structure refinement provides strong evidence that an isostructural compound, originally described as the mixed-valence compound, Ge_2_[H_2_PO_2_]_6_ [Weakley (1983 ▶). *J. Chem. Soc. Pak.* **5**, 279–281], must be reformulated as Ca[Ge(H_2_PO_2_)_6_].

## Related literature

For the reaction of SnF_2_ with H_3_PO_2_, see: Everest (1951 ▶). For the structures of Ge_2_(H_2_PO_2_)_6_, see: Weakley (1983 ▶), and Fe_2_(H_2_PO_2_)_6_, see: Kuratieva & Naumov (2006 ▶). For the so-called Ge_2_(H_2_PO_2_)_6_ structure type, see: Bergerhoff *et al.* (1999 ▶); Villars *et al.* (2010 ▶). For Ca—O bond lengths, see: Smith & Leider (1968 ▶) and for Sn—O bond lengths, see: Yamanaka *et al.* (2000 ▶). Bond-valence sums were calculated using *Valist* (Wills, 2010 ▶)

## Experimental

### 

#### Crystal data

Ca[Sn(H_2_PO_2_)_6_]
*M* *_r_* = 548.69
Hexagonal, 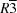

*a* = 11.8619 (4) Å
*c* = 9.8668 (4) Å
*V* = 1202.31 (8) Å^3^
*Z* = 3
Mo *K*α radiation
μ = 2.56 mm^−1^
*T* = 100 K
0.36 × 0.11 × 0.05 mm

#### Data collection

Bruker APEXII CCD diffractometer
Absorption correction: multi-scan (*SADABS*; Bruker, 2009 ▶) *T*_min_ = 0.459, *T*_max_ = 0.887
18525 measured reflections
777 independent reflections
731 reflections with *I* > 2σ(*I*)
*R*_int_ = 0.054

#### Refinement

*R*[*F* ^2^ > 2σ(*F* ^2^)] = 0.020
*wR*(*F* ^2^) = 0.058
*S* = 1.21
777 reflections
33 parameters
H-atom parameters constrained
Δρ_max_ = 1.02 e Å^−3^
Δρ_min_ = −0.42 e Å^−3^

### 

Data collection: *APEX2* (Bruker, 2009 ▶); cell refinement: *SAINT* (Bruker, 2009 ▶); data reduction: *SAINT*; program(s) used to solve structure: *SHELXS97* (Sheldrick, 2008 ▶); program(s) used to refine structure: *SHELXL97* (Sheldrick, 2008 ▶); molecular graphics: *DIAMOND* (Brandenburg, 2006 ▶) and *Mercury* (Macrae *et al.*, 2008 ▶); software used to prepare material for publication: *SHELXTL* (Sheldrick, 2008 ▶).

## Supplementary Material

Crystal structure: contains datablock(s) I, New_Global_Publ_Block. DOI: 10.1107/S1600536813018205/cq2005sup1.cif

Structure factors: contains datablock(s) I. DOI: 10.1107/S1600536813018205/cq2005Isup2.hkl

Additional supplementary materials:  crystallographic information; 3D view; checkCIF report

## Figures and Tables

**Table 1:** Selected bond lengths (Å)

Sn1—O1	2.0266 (15)
Ca1—O2	2.3303 (19)
P1—O2	1.4768 (18)
P1—O1	1.5397 (16)

**Fig. 1.**
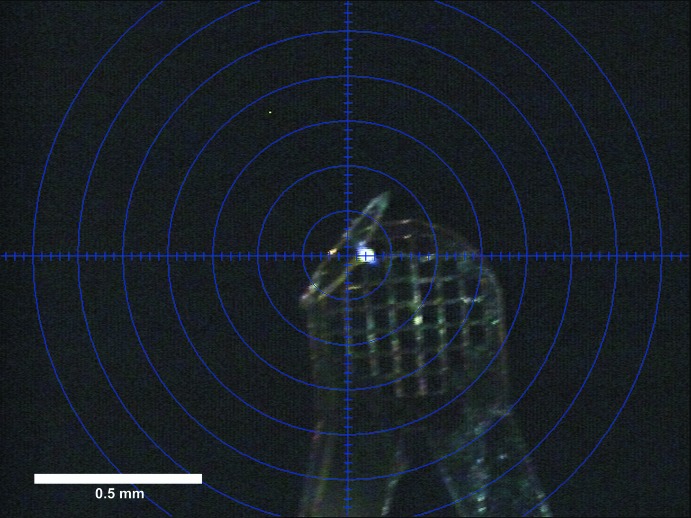
The crystal of Ca[SnH2PO2)6] used for X-ray diffraction measurements showing the crystal shape typical of the title compound.

**Fig. 2.**
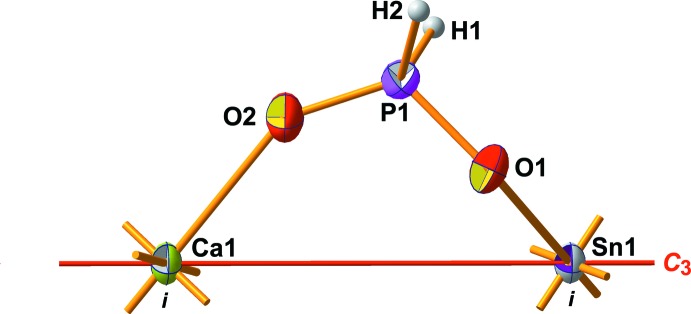
Ball-and-stick model of the asymmetric unit of the title compound with the atomic numbering scheme used. With the exception of the hydrogen atoms, which are shown as spheres with a common isotropic radius, all other atoms are represented as thermal displacement ellipsoids shown at the 50% probability level. Additional bonds from Ca and Sn to oxygen are shown as shortened sticks and the positions of the threefold rotation axis, C3, and inversion centers, i, are also indicated.

**Fig. 3.**
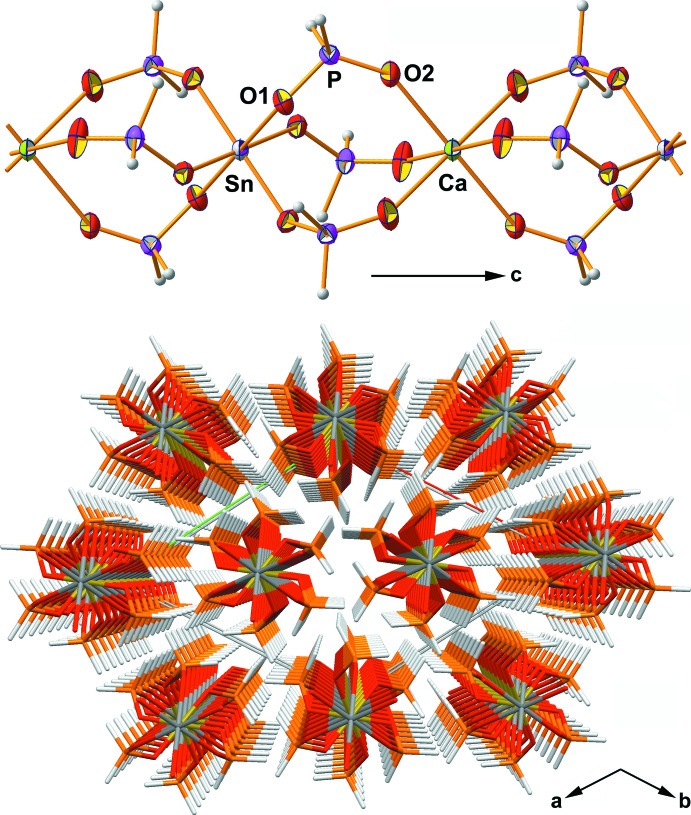
Packing diagram looking down the c axis of the crystal structure of Ca[Sn(H2PO2)6] (below) constructed from hexagonal close packing of the linear chains along [001](above).

**Fig. 4.**
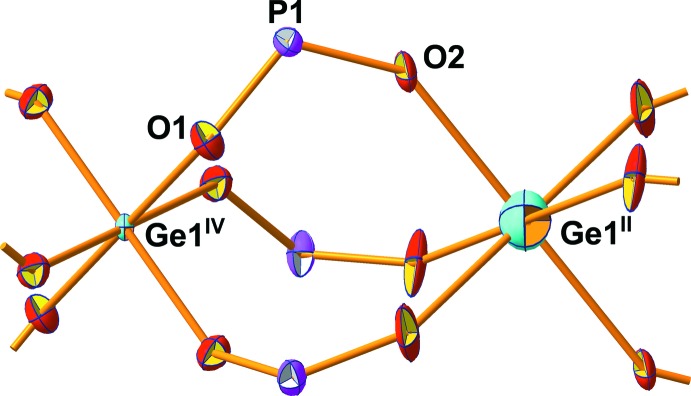
Ball-and-stick model of the asymmetric unit of "Ge2(H2PO2)6" as derived from deposited data (ICSD-37318). All atoms are represented as thermal displacement ellipsoids shown at the 50% probability level. The positions of the hydrogen atoms were not determined.

## supplementary crystallographic information

### Comment

The title compound, Ca[Sn(H_2_PO_2_)_6_], was obtained by chance in the course of the reaction of tin(II)-fluoride, SnF_2_, with phosphinic acid, H_3_PO_2_. According to Everest (1951), a similar reaction of SnCl_2_ with phosphinic acid resulted in the formation of several compounds including mixed chloride hypophosphates of tin(II). In order to simplify the reaction conditions, we studied the reaction in air using small amounts of the reactants and followed the reaction progress by optical microscopy. After a few minutes, SnF_2_ starts to dissolve, accompanied by the growth of clusters of short colourless needles of Sn(H_2_PO_2_)_2_.H_2_O at the surface of the undissolved solid. Within the clear solvent phase around the remaining solid, some large discrete colourless crystals of α-Sn(H_2_PO_2_)_2_ appeared after about 90 minutes while thick discrete shapeless lumps of β-Sn(H_2_PO_2_)_2_ were formed after one day. A day later, a few new crystals had formed within the clear solution with a slightly rounded, rhombohedral shape (Fig.1) that was totally different from the crystal shapes of the compounds formed before. Although the crystals were strongly fixed to the glassware, we were able to isolate a single crystal suitable for X-ray measurements. The unexpected formula Ca[Sn(H_2_PO_2_)_6_] resulted from the structure refinement, indicating that the reaction medium, possibly containing HF formed during the reaction, must have attacked the glassware to generate Ca^2+^ ions and that tin(II) must have been oxidized to tin(IV), presumably by oxygen from air. Bond-valence sums for the tin (4.32) and calcium (2.08) sites, calculated using Valist (Wills, 2010), are in good agreement with the oxidation states of +IV and +II for the respective metal atoms.

The asymmetric unit (Fig. 2) of the title compound consists of 1/6 formula unit with tin and calcium on special positions (3, Wyckoff letter *a* for Sn, and *b* for Ca) linked *via* one hypophosphite ion in a general position. The hypophosphite ion has distorted tetrahedral geometry with an O—P—O angle of 116.1 (1)° and P—O bond lengths which differ by 0.063Å. The shorter P—O bond (P—O2, 1.477 (2) Å] connects to the calcium ion and the longer one (P—O1, 1.540 (2) Å) to the tin(IV) ion.

Because of the high site symmetry of both metal atoms, their coordination polyhedra, formed by six oxygen atoms, are very regular. The six equal Sn—O bond lengths of 2.027 (2) Å are a little bit shorter than the corresponding bonds in SnO_2_ [mean value: 2.055 (2), octahedral coordination] (Yamanaka *et al.*, 2000) as are the six equal Ca—O bond lengths of 2.330 (2) Å in comparison with the corresponding bonds in CaO [2.406 Å, octahedral coordination] (Smith & Leider, 1968). Angle distortions of the octahedra in the present compound are about ∓ 1.5° at tin, and ∓ 1.1° at calcium.

Because both metal atoms are situated on the same threefold rotation axis, they are arranged in linear chains along the [001] direction, resulting in strands that are perfectly hexagonal closed packed (Fig. 3). Interchain interactions are restricted to van der Waals' ones.

The title compound is isostructural with Fe_2_(H_2_PO_2_)_3_ (Kuratieva & Naumov, 2006) and Ge_2_(H_2_PO_2_)_6_ (Weakley, 1983), the latter compound being the prototype of the so-called "Ge_2_(H_2_PO_2_)_6_" structure type (Bergerhoff *et al.*, 1999; Villars *et al.*, 2010). In Fe_2_(H_2_PO_2_)_3_, both Fe ions are in the same oxidation state, +III, with very similar coordination parameters for both, whereas the later should be a mixed-valence germanium compound with the Ge ions in oxidation states +II and +IV. In the latter case, the bond lengths for both Ge ions are different: 1.869 (8) Å for Ge(IV) and 2.322 (9) Å for Ge(II). In contrast to the situation in other Ge(II) compounds, where the lone pair of electrons are sterically active giving rise to a strongly distorted coordination behaviour, the coordination polyhedron of the Ge(II) in Ge_2_(H_2_PO_2_)_6_ is a very regular octahedron with bond angles ranging from 88.2 (3)° to 91.8 (3)°. The unexpected stereochemical behaviour of this Ge(II) ion in the title compound, and the observation that the Ge(II)—O bond lengths are nearly identical (at 2.322 (9) Å) to those of Ca—O (at 2.330 (2) Å), lead us to suggest that the assignment of this atom site as Ge(II) must be incorrect and should be Ca(II) instead. Some further hints resulting from chemical and crystallographical information strengthen the suggestion that Ge_2_(H_2_PO_2_)_6_ is better described as Ca[Ge(H_2_PO_2_)_6_]. For example, as in case of the title compound, the formation of Ge(H_2_PO_2_)_6_ was not the result of a rational synthesis, but was formed as a minor product during the reaction of a dihalide of germanium with phosphinic acid. Furthermore, the anisotropic thermal displacement factors of the atom assigned to Ge(II) are much higher than those of Ge(IV) (Fig. 4) indicating that the electron density at this site must be significantly lower than for Ge(II). It would be interesting to recalculate the structure using Ca(II) instead of Ge(II) in order to verify this suggestion and to lower the high *R*-value (9.3%), but no intensity data are deposited, unfortunately.

### Experimental

About 100 milligram of solid tin(II)-fluoride (Sigma-Aldrich) were placed on a petri dish and covered by a few drops of phosphinic acid (50%, Sigma-Aldrich). No special precautions were taken to exclude air. The progress of dissolution and crystal formation was followed by optical microscopy. A few crystals of the title compound were formed after some days within the clear solution. All attempts to prepare the compound in a larger quantity in order to perform elemental analysis have so far been unsuccessful. A suitable single crystal was mounted on a 50 µm MicroMesh MiTeGen Micromount^TM^ using Fromblin Y perfluoropolyether (LVAC 16/6, Aldrich).

### Refinement

Because the composition of the title compound was unknown, structure solution started using the formula of a mixed fluoride hypophosphate of tin(II). From Direct Methods, a tin atom on a special position as well as a PO_2_ fragment on a general position could be determined unambiguously. The identification of another atom on a second special position was much more difficult. Several tests using different elements during the structure refinement were unsuccessful but convinced us that the tin atom must be in oxidation state +IV (and not +II as first assumed ) with an additional divalent cation at the second special position to counterbalance the negative charge of the [H_2_PO_2_]^-^ ions. In fact, placing a tin(II) atom on the position in question improved the structure refinement to some extent, but the large isotropic displacement parameter indicated that electron density at this site should be much smaller. Further tests with other divalent cations instead of tin(II) gave the best result in case of Ca^2+^.

At this stage of refinement H-atoms could be localized in difference Fourier synthesis. Their positions were refined with respect to a P—H distance of 1.39 Å before they were fixed and allowed to ride on the phosphorus atom with a common isotropic displacement factor.

### Crystal data

**Table:**

Ca[Sn(H_2_PO_2_)_6_]	*D*_x_ = 2.273 Mg m^−^^3^
*M**_r_* = 548.69	Mo *K*α radiation, λ = 0.71073 Å
Hexagonal, *R*3	Cell parameters from 6570 reflections
Hall symbol: -R 3	θ = 2.9–30.0°
*a* = 11.8619 (4) Å	µ = 2.56 mm^−^^1^
*c* = 9.8668 (4) Å	*T* = 100 K
*V* = 1202.31 (8) Å^3^	Needle, colourless
*Z* = 3	0.36 × 0.11 × 0.05 mm
*F*(000) = 804	

### Data collection

**Table:**

Bruker APEXII CCD diffractometer	777 independent reflections
Radiation source: fine-focus sealed tube	731 reflections with *I* > 2σ(*I*)
Graphite monochromator	*R*_int_ = 0.054
φ and ω scans	θ_max_ = 30.0°, θ_min_ = 3.4°
Absorption correction: multi-scan (*SADABS*; Bruker, 2009)	*h* = −16→16
*T*_min_ = 0.459, *T*_max_ = 0.887	*k* = −16→16
18525 measured reflections	*l* = −13→13

### Refinement

**Table:**

Refinement on *F*^2^	Primary atom site location: structure-invariant direct methods
Least-squares matrix: full	Secondary atom site location: difference Fourier map
*R*[*F*^2^ > 2σ(*F*^2^)] = 0.020	Hydrogen site location: inferred from neighbouring sites
*wR*(*F*^2^) = 0.058	H-atom parameters constrained
*S* = 1.21	*w* = 1/[σ^2^(*F*_o_^2^) + (0.0229*P*)^2^ + 3.9531*P*] where *P* = (*F*_o_^2^ + 2*F*_c_^2^)/3
777 reflections	(Δ/σ)_max_ = 0.001
33 parameters	Δρ_max_ = 1.02 e Å^−^^3^
0 restraints	Δρ_min_ = −0.42 e Å^−^^3^

### Special details

**Table:**

Geometry. All e.s.d.'s (except the e.s.d. in the dihedral angle between two l.s. planes) are estimated using the full covariance matrix. The cell e.s.d.'s are taken into account individually in the estimation of e.s.d.'s in distances, angles and torsion angles; correlations between e.s.d.'s in cell parameters are only used when they are defined by crystal symmetry. An approximate (isotropic) treatment of cell e.s.d.'s is used for estimating e.s.d.'s involving l.s. planes.
Refinement. Refinement of *F*^2^ against ALL reflections. The weighted *R*-factor *wR* and goodness of fit *S* are based on *F*^2^, conventional *R*-factors *R* are based on *F*, with *F* set to zero for negative *F*^2^. The threshold expression of *F*^2^ > σ(*F*^2^) is used only for calculating *R*-factors(gt) *etc*. and is not relevant to the choice of reflections for refinement. *R*-factors based on *F*^2^ are statistically about twice as large as those based on *F*, and *R*- factors based on ALL data will be even larger.

### Fractional atomic coordinates and isotropic or equivalent isotropic displacement parameters (Å^2^)

**Table:**

	*x*	*y*	*z*	*U*_iso_*/*U*_eq_	
Sn1	1.0000	1.0000	0.0000	0.01807 (11)	
Ca1	1.0000	1.0000	0.5000	0.0192 (2)	
P1	0.78540 (5)	0.97330 (6)	0.22095 (6)	0.02205 (14)	
H1	0.7214	0.8488	0.1657	0.040 (6)*	
H2	0.7073	1.0296	0.2122	0.040 (6)*	
O1	0.89193 (16)	1.05182 (16)	0.11547 (15)	0.0244 (3)	
O2	0.82943 (19)	0.9728 (2)	0.36084 (18)	0.0408 (5)	

### Atomic displacement parameters (Å^2^)

**Table:**

	*U*^11^	*U*^22^	*U*^33^	*U*^12^	*U*^13^	*U*^23^
Sn1	0.02121 (13)	0.02121 (13)	0.01179 (16)	0.01061 (7)	0.000	0.000
Ca1	0.0229 (3)	0.0229 (3)	0.0117 (4)	0.01146 (15)	0.000	0.000
P1	0.0205 (3)	0.0291 (3)	0.0184 (3)	0.0138 (2)	0.00185 (19)	0.0010 (2)
O1	0.0300 (8)	0.0253 (8)	0.0196 (7)	0.0150 (7)	0.0060 (6)	0.0019 (6)
O2	0.0325 (10)	0.0739 (15)	0.0187 (8)	0.0286 (10)	0.0021 (7)	0.0070 (8)

### Geometric parameters (Å, º)

**Table:**

Sn1—O1^i^	2.0265 (15)	Ca1—O2^ii^	2.3302 (19)
Sn1—O1^ii^	2.0265 (15)	Ca1—O2^viii^	2.3302 (19)
Sn1—O1^iii^	2.0266 (16)	Ca1—O2^iv^	2.3302 (19)
Sn1—O1^iv^	2.0266 (16)	Ca1—O2	2.3303 (19)
Sn1—O1	2.0266 (15)	P1—O2	1.4768 (18)
Sn1—O1^v^	2.0266 (16)	P1—O1	1.5397 (16)
Ca1—O2^vi^	2.3302 (19)	P1—H1	1.3900
Ca1—O2^vii^	2.3302 (19)	P1—H2	1.3900
			
O1^i^—Sn1—O1^ii^	180.0	O2^vii^—Ca1—O2^viii^	88.81 (7)
O1^i^—Sn1—O1^iii^	91.49 (6)	O2^ii^—Ca1—O2^viii^	91.19 (7)
O1^ii^—Sn1—O1^iii^	88.51 (6)	O2^vi^—Ca1—O2^iv^	91.19 (7)
O1^i^—Sn1—O1^iv^	88.51 (6)	O2^vii^—Ca1—O2^iv^	91.19 (7)
O1^ii^—Sn1—O1^iv^	91.49 (6)	O2^ii^—Ca1—O2^iv^	88.81 (7)
O1^iii^—Sn1—O1^iv^	180.00 (11)	O2^viii^—Ca1—O2^iv^	180.0
O1^i^—Sn1—O1	88.52 (6)	O2^vi^—Ca1—O2	180.0
O1^ii^—Sn1—O1	91.48 (6)	O2^vii^—Ca1—O2	91.19 (7)
O1^iii^—Sn1—O1	88.52 (6)	O2^ii^—Ca1—O2	88.81 (7)
O1^iv^—Sn1—O1	91.48 (6)	O2^viii^—Ca1—O2	91.19 (7)
O1^i^—Sn1—O1^v^	91.48 (6)	O2^iv^—Ca1—O2	88.81 (7)
O1^ii^—Sn1—O1^v^	88.52 (6)	O2—P1—O1	116.70 (11)
O1^iii^—Sn1—O1^v^	91.48 (6)	O2—P1—H1	111.6
O1^iv^—Sn1—O1^v^	88.52 (6)	O1—P1—H1	103.0
O1—Sn1—O1^v^	180.0	O2—P1—H2	112.5
O2^vi^—Ca1—O2^vii^	88.81 (7)	O1—P1—H2	102.1
O2^vi^—Ca1—O2^ii^	91.19 (7)	H1—P1—H2	110.1
O2^vii^—Ca1—O2^ii^	180.0	P1—O1—Sn1	130.45 (10)
O2^vi^—Ca1—O2^viii^	88.81 (7)	P1—O2—Ca1	146.56 (12)

Symmetry codes: (i) *y*, −*x*+*y*+1, −*z*; (ii) −*y*+2, *x*−*y*+1, *z*; (iii) *x*−*y*+1, *x*, −*z*; (iv) −*x*+*y*+1, −*x*+2, *z*; (v) −*x*+2, −*y*+2, −*z*; (vi) −*x*+2, −*y*+2, −*z*+1; (vii) *y*, −*x*+*y*+1, −*z*+1; (viii) *x*−*y*+1, *x*, −*z*+1.
